# Recurrent Spontaneous Coronary Artery Dissection (SCAD) With a Family History of Cardiovascular Disease: A Case Report

**DOI:** 10.7759/cureus.96029

**Published:** 2025-11-03

**Authors:** Carolina Suárez Burneo, Pablo X Barzallo

**Affiliations:** 1 Health Sciences, Universidad Técnica Particular de Loja, Loja, ECU; 2 Cardiology, University of Illinois College of Medicine Peoria, Peoria, USA

**Keywords:** acute coronary syndrome, chest pain, family medical history, fibromuscular dysplasia, spontaneous coronary artery dissection

## Abstract

Spontaneous coronary artery dissection (SCAD) is a rare cause of acute coronary syndrome and is often associated with fibromuscular dysplasia (FMD). We describe the case of a 55-year-old woman with a prior history of SCAD who presented with acute chest pain. Angiography revealed a new dissection in the diagonal branch of the left anterior descending artery with preserved flow, but her condition worsened with recurrent pain and ischemic electrocardiographic changes, ultimately requiring intra-aortic balloon pump support. She stabilized and was discharged on medical therapy, with plans for further evaluation of possible FMD. This case highlights the clinical complexity of recurrent SCAD, the importance of considering family history in risk assessment, and the challenges of management, particularly regarding revascularization strategies versus conservative therapy. Given the association between SCAD and FMD, screening for underlying arteriopathies is crucial. Recognition of recurrent SCAD in patients with few traditional cardiovascular risk factors is essential for timely diagnosis, appropriate management, and risk stratification.

## Introduction

Spontaneous coronary artery dissection (SCAD) is an increasingly recognized non-atherosclerotic cause of acute coronary syndrome (ACS). Although it accounts for only 1-4% of ACS cases overall, SCAD is responsible for up to 35% of myocardial infarctions in women under 50 and is the leading cause of pregnancy-associated myocardial infarction [1]. SCAD is defined as the occlusion of the lumen of a coronary artery secondary to an intramural hematoma and intimal disruption, in the absence of atherosclerosis or trauma [1]. These vascular changes can result in ACS, arrhythmia, or sudden cardiac death, most frequently affecting young and middle-aged women [2]. Possible triggers include pregnancy, emotional or physical stress, genetic predisposition, and hormonal influences [3].

Fibromuscular dysplasia (FMD) is a non-atherosclerotic arteriopathy characterized by abnormal cellular proliferation and distortion of the arterial wall [4]. A strong association has been reported between FMD and SCAD, particularly in women with few traditional cardiovascular risk factors. Concomitant FMD has been documented in up to 86% of SCAD patients, supporting a possible shared pathophysiological or genetic basis, and intracoronary imaging suggests that SCAD may represent the first clinical manifestation of coronary FMD [5].

Recurrence is an important clinical consideration in SCAD. Long-term studies indicate that up to one-third of patients experience recurrent events within 10 years, with recurrence rates ranging from 10% to 17% at three years [5].

Despite increasing awareness, SCAD remains underdiagnosed and its optimal management continues to be debated. Evidence suggests that genetic and familial factors may contribute to the pathogenesis of SCAD, particularly in patients with concomitant FMD or recurrent events, highlighting a potential heritable vascular predisposition [3,5]. Here, we report the case of a 55-year-old woman with recurrent SCAD, a strong family history of coronary events, and evaluation for underlying FMD.

## Case presentation

A 55-year-old woman with a past medical history of SCAD in 2017 underwent left heart catheterization (LHC) for a presumed non-ST-elevation myocardial infarction (NSTEMI). The procedure revealed a spontaneous dissection of the mid/distal left anterior descending artery (LAD) with preserved flow. She was discharged on dual antiplatelet therapy (DAPT) consisting of aspirin (81 mg), clopidogrel (75 mg), and a beta-blocker (metoprolol tartrate, 25 mg).

Several years after her initial SCAD diagnosis, the patient presented to the emergency department with central chest pain lasting 2.5 hours, described as a pressure-like sensation radiating to the back and resembling her prior SCAD event. The pain was associated with nausea and diaphoresis. It initially improved after two sublingual doses of nitroglycerin but recurred shortly thereafter. On arrival, the patient was alert and oriented. Neck examination revealed no jugular venous distension. Cardiac auscultation demonstrated a regular rhythm with normal S1 and S2, without murmurs. Lungs were clear bilaterally, with no wheezes, rales, or crackles. No peripheral edema was noted.

**Figure 1:**
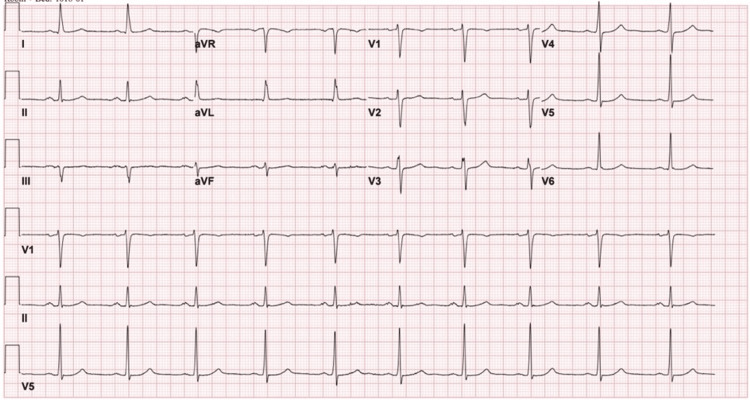
ECG prior LHC. Regular sinus rhythm with bradycardia. LHC: left heart catheterization.

In addition to her prior SCAD, she had a history of essential hypertension (HTN) and hypercholesterolemia. Her family history was significant for early-onset coronary events: a brother who experienced a myocardial infarction (MI) at 29 years, a father who had MIs in his 40s, and a mother with a history of SCAD. She had been a smoker for 10 years prior to cessation. Upon admission, the initial laboratory tests showed elevated troponin and no relevant changes on ECG besides sinus bradycardia (Figure 1). Initial troponin levels were elevated at 31 ng/L, peaking at 143 ng/L (reference range <14 ng/L in females).

**Figure 2:**
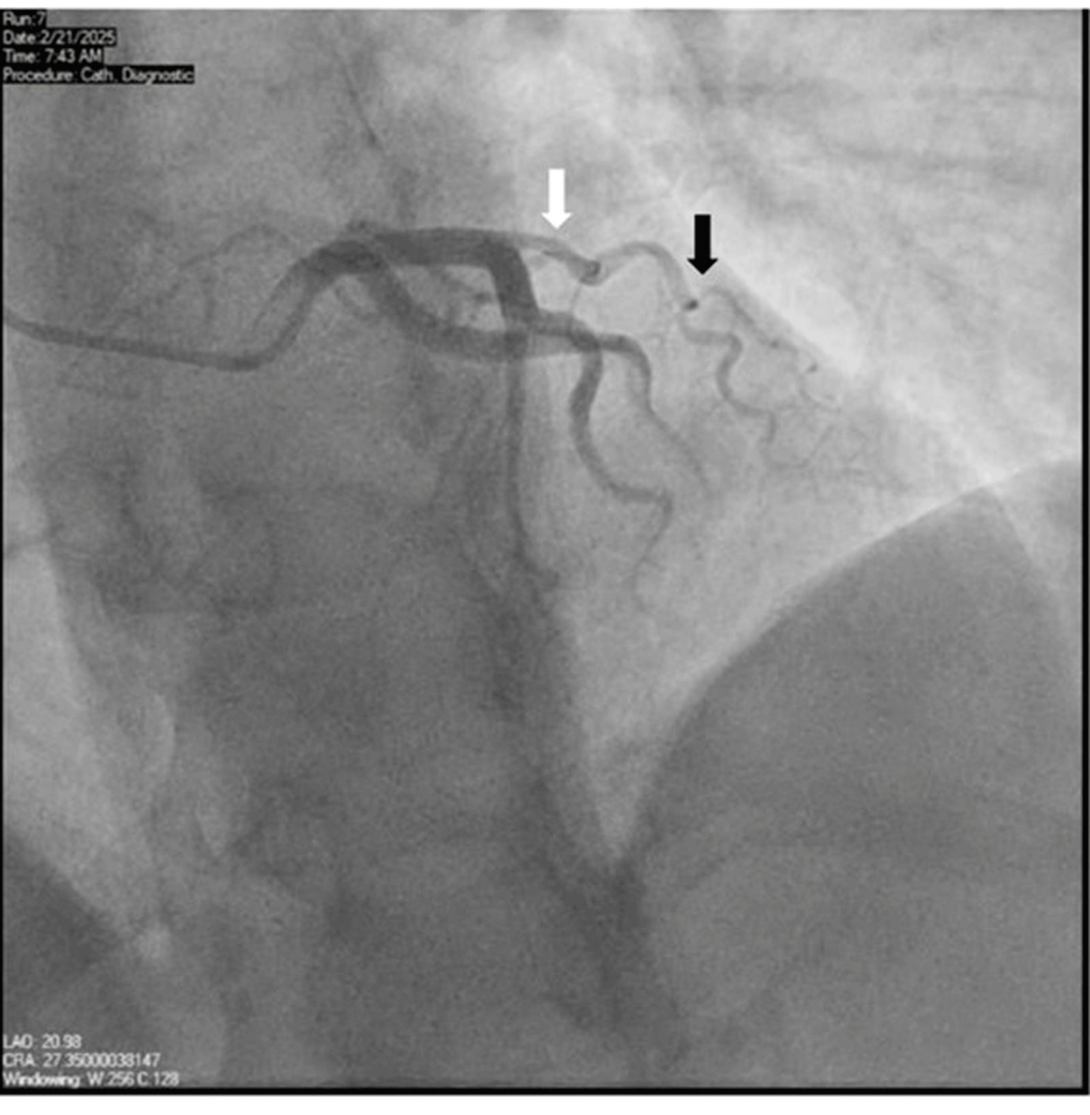
Left coronary vessel showing a proximal vessel lesion on the diagonal branch (black arrow) in addition to the prior SCAD on the mid-LAD, which is healed (white arrow). LAD: left anterior descending artery; SCAD: spontaneous coronary artery dissection.

She underwent LHC, which revealed a right-dominant circulation with no significant atherosclerosis. The previously identified SCAD in the mid-LAD appeared to have healed. However, a new spontaneous dissection was suspected in the diagonal branch of the apical LAD, with preserved TIMI 3 flow (Figure 2). Medical management was initially recommended.

**Figure 3:**
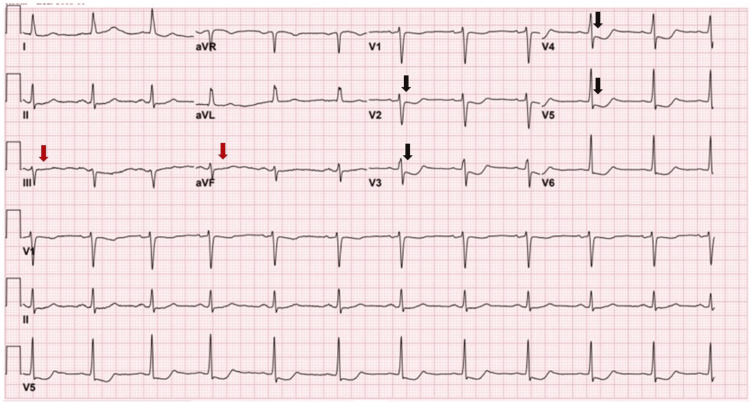
ECG post LHC, showing ST depressions on leads V2 –V5 with T-wave inversions (black arrows). Also, there are nonspecific T-wave abnormalities on leads III and aVF (red arrows). LHC: left heart catheterization.

Later that day, after the LHC, the patient reported severe chest pain. An ECG showed new ST depression in the anterior leads, nonspecific T-wave abnormalities in the inferior leads, and new T-wave inversion in the anterior leads (Figure 3).

**Figure 4:**
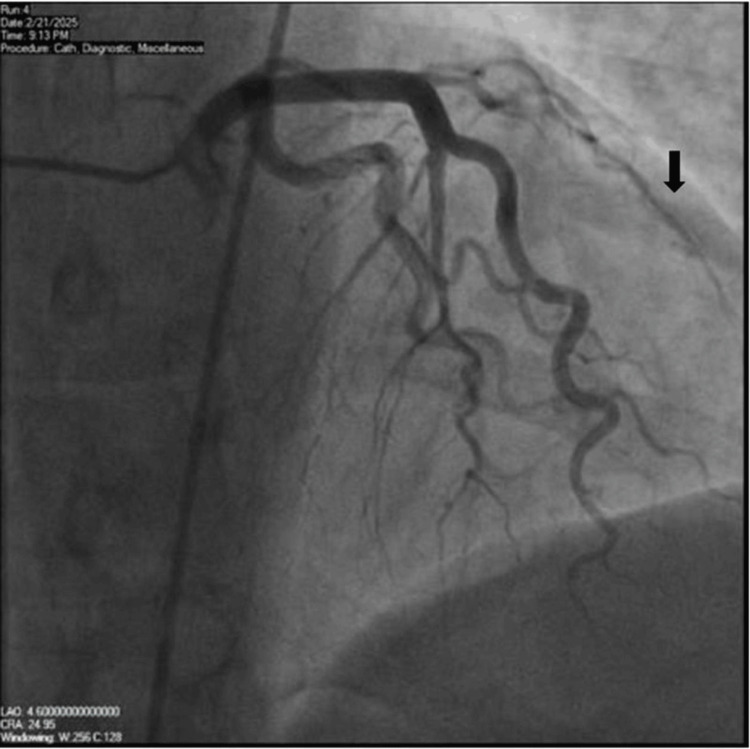
Left coronary vessel showing a dissection on the diagonal branch, extending through the whole artery (black arrow).

A repeat LHC on the same day demonstrated worsening dissection of the diagonal branch, with reduced TIMI flow (0-1) (Figure 4). This prompted the placement of an intra-aortic balloon pump with 1:1 augmentation, resulting in immediate and complete resolution of chest pain (pain score decreased from 10 to 0).

Following intra-aortic balloon pump placement, the patient’s chest pain resolved completely. Troponin levels trended downward, and subsequent ECGs demonstrated resolution of ischemic changes. A transthoracic echocardiogram (TTE) performed two days after the balloon pump placement demonstrated mild septal motion abnormality due to a conduction delay and a trivial pericardial effusion with no significant change from the prior study. The left ventricular ejection fraction (LVEF) was visually estimated at 55%.

Given the strong family history of early coronary events and SCAD, a workup for FMD was initiated. Carotid duplex ultrasound showed no significant stenosis. A renal ultrasound was planned but could not be completed during the admission. The patient was discharged pain-free on aspirin 81 mg and a beta blocker (metoprolol succinate). Dual antiplatelet therapy was not indicated given the nature of the coronary dissection.

The patient was referred to a specialist for further evaluation of underlying connective tissue disorders and potential FMD. She was advised to avoid strenuous activity and heavy lifting for six weeks. Follow-up with a cardiologist was scheduled, with the option of referral to a tertiary center for further evaluation.

## Discussion

SCAD predominantly affects middle-aged women, with a reported prevalence of 87-95% in this population [3]. The LAD is involved in most cases (>50%) [6]. Despite a lower prevalence of traditional cardiovascular risk factors, hyperlipidemia and hypertension are present in approximately 30% of patients [3].

SCAD and FMD

Several studies have demonstrated an association between SCAD and FMD. Both conditions primarily affect middle-aged women and are frequently underdiagnosed. The prevalence of FMD in patients with SCAD exceeds 50% [7]. FMD has been identified as a risk factor for recurrence and is associated with a higher risk of major adverse cardiovascular events (MACE) [6].

A higher incidence of familial FMD has been observed in patients with arterial dissections. The intronic PHACTR1 variant has been identified in both FMD and SCAD. Nevertheless, other common genetic variants differ between FMD and SCAD, and polygenic risk scores for SCAD can predict the risk of SCAD in patients with FMD. Thus, although the two conditions share a gene locus (PHACTR1), additional genetic risk factors are involved [8]. These findings support the emerging concept of “familial SCAD,” suggesting that genetic testing, particularly for variants such as PHACTR1, may help identify individuals with an inherited predisposition to these conditions.

Management

Coronary angiography is the first-line diagnostic tool for patients with ACS suspected to be caused by SCAD. Additional diagnostic modalities may be indicated depending on SCAD types 1-3 [3].

Management varies depending on the severity of the lesion and the patient’s hemodynamic status. In most cases, a conservative treatment strategy is recommended, followed by an inpatient monitoring period of three to five days [3]. Long-term therapy also varies. In patients undergoing percutaneous coronary intervention (PCI), dual antiplatelet therapy (DAPT) is recommended [1]. In this case, for non-PCI patients, the benefit of treatment with DAPT is unclear. Some guidelines recommend DAPT for one to three months, followed by at least one year with aspirin or as a long-term monotherapy [5]. Additionally, the use of beta-blockers has been associated with a lower recurrence of SCAD [5].

Recent studies have identified new associations regarding DAPT in SCAD management. The combination of aspirin and ticagrelor (P2Y12) is associated with a higher risk of SCAD recurrence, with a 1.8-fold increase in the risk of MACE, whereas this risk is not observed with clopidogrel [6].

An important aspect of management is screening for FMD. The most commonly affected arteries are the renal, cerebrovascular, and carotid arteries [4]. Computed tomographic angiography (CTA) and duplex ultrasound of the renal and carotid arteries are the initial tests of choice for screening [9].

Recurrence risk remains significant in the first years following SCAD [5]. This underscores the importance of long-term follow-up, patient education on symptom recognition, and lifestyle counseling. Moreover, psychological support is crucial, as anxiety related to recurrence is frequently reported among SCAD survivors [10].

## Conclusions

In young female patients with a history of SCAD, particularly those with a strong family history or recurrent events, screening for FMD should be incorporated into the management plan. Additionally, conservative pharmacological therapy should be carefully tailored, as certain DAPT combinations, such as aspirin and ticagrelor, may increase the risk of SCAD recurrence. In this case, FMD was suspected but not confirmed, highlighting the importance of comprehensive vascular evaluation and long-term follow-up. Future research exploring genetic predisposition, optimal pharmacologic strategies, and the long-term psychosocial outcomes of SCAD survivors will be essential to improve prognosis and guide personalized care.

## References

[1] Hayes SN, Kim ES, Saw J (2018). Spontaneous coronary artery dissection: current state of the science: a scientific statement from the American Heart Association. Circulation.

[2] Matta A, Levai L, Elbaz M, Nader V, Parada FC, Carrié D, Roncalli J (2023). Spontaneous coronary artery dissection: a review of epidemiology, pathophysiology and principles of management. Curr Probl Cardiol.

[3] Hayes SN, Tweet MS, Adlam D, Kim ES, Gulati R, Price JE, Rose CH (2020). Spontaneous coronary artery dissection: JACC state-of-the-art review. J Am Coll Cardiol.

[4] Gornik HL, Persu A, Adlam D (2019). First International Consensus on the diagnosis and management of fibromuscular dysplasia. Vasc Med.

[5] Shah T, Zhong M, Feldman DN (2025). Spontaneous coronary artery dissection: diagnostic challenges and updates in management. Curr Treat Options Cardiovasc Med.

[6] Dang QM, Psaltis PJ, Burgess S (2025). The Australian-New Zealand spontaneous coronary artery dissection cohort study: predictors of major adverse cardiovascular events and recurrence. Eur Heart J.

[7] Kim ES, Saw J, Kadian-Dodov D, Wood M, Ganesh SK (2021). FMD and SCAD: sex-biased arterial diseases with clinical and genetic pleiotropy. Circ Res.

[8] Huart J, Stoenoiu MS, Zedde M, Pascarella R, Adlam D, Persu A (2023). From fibromuscular dysplasia to arterial dissection and back. Am J Hypertens.

[9] O'Connor SC, Gornik HL (2014). Recent developments in the understanding and management of fibromuscular dysplasia. J Am Heart Assoc.

[10] Johnson AK, Hayes SN, Sawchuk C, Johnson MP, Best PJ, Gulati R, Tweet MS (2020). Analysis of posttraumatic stress disorder, depression, anxiety, and resiliency within the unique population of spontaneous coronary artery dissection survivors. J Am Heart Assoc.

